# Methicillin-Resistant *Staphylococcus aureus* Blood Isolates Harboring a Novel Pseudo-staphylococcal Cassette Chromosome *mec* Element

**DOI:** 10.3389/fmicb.2019.00540

**Published:** 2019-03-29

**Authors:** Eun-Jeong Yoon, Hyukmin Lee, Dokyun Kim, Jong Hee Shin, Jeong Hwan Shin, Seok Hoon Jeong

**Affiliations:** ^1^ Department of Laboratory Medicine, Research Institute of Bacterial Resistance, Yonsei University College of Medicine, Seoul, South Korea; ^2^ Department of Laboratory Medicine, Chonnam National University Medical School, Gwangju, South Korea; ^3^ Department of Laboratory Medicine, Paik Institute for Clinical Research, Inje University College of Medicine, Busan, South Korea

**Keywords:** methicillin-resistant *Staphylococcus aureus*, sequence type 72, pseudo-SCC*mec*, *ccr* gene, blood isolates

## Abstract

The aim of this work was to assess a novel pseudo-staphylococcal cassette chromosome *mec* (ΨSCC*mec*) element in methicillin-resistant *Staphylococcus aureus* (MRSA) blood isolates. Community-associated MRSA E16SA093 and healthcare-associated MRSA F17SA003 isolates were recovered from the blood specimens of patients with *S. aureus* bacteremia in 2016 and in 2017, respectively. Antimicrobial susceptibility was determined *via* the disk diffusion method, and SCC*mec* typing was conducted by multiplex polymerase chain reaction. Whole genome sequencing was carried out by single molecule real-time long-read sequencing. Both isolates belonged to sequence type 72 and *agr*-type I, and they were negative for Panton-Valentine leukocidin and toxic shock syndrome toxin. The *spa*-types of E16SA093 and F17SA003 were t324 and t2460, respectively. They had a SCC*mec* IV-like element devoid of the cassette chromosome recombinase (*ccr*) gene complex, designated as ΨSCC*mec*_E16SA093_. The element was manufactured from SCC*mec* type IV and the deletion of the *ccr* gene complex and a 7.0- and 31.9-kb portion of each chromosome. The deficiency of the *ccr* gene complex in the SCC*mec* unit is likely resulting in mobility loss, which would be an adaptive evolutionary mechanism. The dissemination of this clone should be monitored closely.

## Introduction

Methicillin-resistant *Staphylococcus aureus* (MRSA) clinical isolates were first identified in the early 1960s, immediately after the introduction of penicillinase-stable penicillins in the clinical setting (Jevons et al., 1963). Now, the spread of MRSA strains represents a global concern with a recognizable healthcare burden. The *mecA* and *mecC* genes encoding penicillin-binding protein (PBP) 2a of low beta-lactam binding affinity confer beta-lactam resistance to the bacterial host by composing a mobile genetic element, namely, the staphylococcal cassette chromosome *mec* (SCC*mec*) (Ito et al., 1999).

The SCC*mec* element harbors two fundamental components: a *mec* gene complex and a cassette chromosome recombinase (*ccr*) gene complex. A unique combination of the *mec* gene complex class and the *ccr* gene complex allotype determines the type of the SCC*mec* element, and its variation within the joining- (J-) regions determine the subtypes of each SCC*mec* type. To date, 13 SCC*mec* types have been deposited together with numerous subtypes (International Working Group on the Classification of Staphylococcal Cassette Chromosome, 2009; Baig et al., 2018). The *mec* gene complex includes the *mecA* or *mecC* gene, along with the regulatory *mecR1* and *mecI* genes. The *ccr* gene complex comprises one or more *ccr* genes (Deurenberg and Stobberingh, 2008) accounting for the integration/excision of the SCC*mec* element into and out of the *orfX* gene in the staphylococcal chromosome (Katayama et al., 2000). The SCC*mec*-like elements devoid of the *mecA* gene are denominated as an SCC as long as they share the following characteristics with SCC*mec*: carriage of the *ccr* gene(s), integration at integrated site sequences in the chromosome, and the presence of flanking direct repeat sequences. And those without the *ccr* genes are termed as the pseudo-(Ψ) SCC*mec* element.

Through a nationwide antimicrobial resistance surveillance in South Korea (Lee et al., 2018), two *mecA*-positive MRSA blood isolates were identified as those carrying a non-typeable SCC*mec* element. To assess the non-typeable SCC*mec*, both genomes were entirely sequenced, and a novel ΨSCC*mec*_E16SA093_ element was identified.

## Materials and Methods

### Bacterial Isolates

A total of 586 *S. aureus* blood isolates collected between May 2016 and April 2017 from six general hospitals in South Korea (Lee et al., 2018) were screened. Among the 319 cefoxitin-resistant isolates, E16SA093 and F17SA003, whose SCC*mec* elements could not be typified, were selected for further study.

### Antimicrobial Susceptibility Testing and the Determination of SCC*mec* Types

Antimicrobial susceptibility to antimicrobials used for staphylococci infection was determined by disk diffusion tests on Mueller-Hinton agar (Difco Laboratories, Detroit, MI, USA), following the CLSI guidelines (CLSI, 2018). *S. aureus* ATCC 25923 was simultaneously tested in each batch for quality control. MRSA isolates were subjected to polymerase chain reaction (PCR) for *mecA* gene and SCC*mec* typing, as previously described (Oliveira and de Lencastre, 2002).

### Multilocus Sequence Typing, *agr* Typing, and *spa* Typing

Multilocus sequence typing (MLST) was carried out by PCR and sequencing of the seven housekeeping genes, *arcC*, *aroE*, *glpF*, *gmk*, *pta*, *tpi*, and *yqiL*. Allelic numbers and sequence types (STs) were determined by comparing the obtained sequences to the database for *S. aureus*^1^. The *agr* type was determined by multiplex PCR (Gilot et al., 2002), and *spa* typing was conducted by comparing the PCR-amplified nucleotide sequence of the variable repeat region of the *spa* gene against the Ridom SpaServer^2^.

### Whole Genome Sequencing

Bacterial whole genomes were sequenced with single-molecule real-time (SMRT) sequencing on a PacBio RSII instrument (Pacific Biosciences, Menlo Park, CA, USA) using genomic DNA from the *S. aureus* isolates extracted by a Wizard Genomic DNA Purification kit (Promega, Madison, WI, USA). SMRTbell template libraries were prepared, and adapter ligation was performed. Acquired sequencing data were *de novo* assembled by PacBio SMRT, read with the PacBio SMRT analysis software suite (version 2.3.0). Coding sequences (CDS), including tRNA and rRNA, were annotated using the NCBI Prokaryotic Genome Annotation Pipeline^3^. Nucleic acid sequences were compared using Basic Local Alignment Search Tool,^4^ and resistance and virulence determinants were searched for using ResFinder^5^ and VirulenceFinder^6^, respectively. Prophages were searched for using the PHAge Search Tool Enhanced Release^7^. For any putative *ccr* gene, the site-specific serine recombinase motif (Wang and Archer, 2010) and a modified motif by the consensus pattern (Perreten et al., 2013) were searched for against the coding sequences of both genomes.

### Nucleotide Accession Numbers

The nucleotide sequences of the entire genomes of *S. aureus* E16SA093 and F17SA003 were deposited in GenBank under accession numbers CP031130 and CP031131 for F17SA003 and E16SA093, respectively.

## Results and Discussion

### Epidemiological Features of MRSA ST72

Following the one-year collection of the 586 *S. aureus* blood isolates, a total of 319 isolates (54.4%) were MRSA conferred by the *mecA* gene. A total of 176 (30.0%) isolates belonged to ST72; 112 of those isolates (63.6%) were MRSA, 65 were healthcare-associated (HA) MRSA, and 47 were community-associated MRSA (CA-MRSA). All but three MRSA ST72 isolates (97.3%, 109/112) carried SCC*mec* type IV, while one possessed SCC*mec* type II and the remaining two isolates (E16SA093 and F17SA003) had non-typeable elements.

The MRSA ST72 harboring SCC*mec* IV (ST72-MRSA-IV) was one of the top three CA-MRSA clones in the USA until 2002 as a pulse-field type USA700; however, the clone was suddenly eliminated from the country in 2004 (Tenover et al., 2008). In South Korea, the ST72-MRSA-IV acceded a major CA-MRSA clone by 2005 (Kim et al., 2007), and the ST72-MRSA-IV subsequently grew to be a major HA-MRSA clone. This finding supports the idea that the ST72-MRSA-IV clone was disseminating from communities to hospitals (Song et al., 2011).

### Two *mecA*-Positive MRSA ST72 Blood Isolates Carrying a Non-typeable SCC*mec* Element

The CA-MRSA E16SA093 was recovered in September 2016 from an 86-year-old female patient with acute infective endocarditis and infective spondylopathy. The patient was transferred from an acute care hospital to a general hospital located in Gwangju city, and blood cultures were carried straightaway. The bacteremia originated from a bone infection, and empirical treatment was started with cefazoline. Definitive treatment was followed with teicoplanin within 72 h after the initial blood culture, and the patient was cured. The HA-MRSA 17SA003 was recovered in January 2017 from a 63-year-old male patient with diabetes mellitus and stage 4 chronic kidney disease hospitalized in a general hospital in Busan city. An initial blood culture was performed on the 13th day of hospitalization, and the origin of *S. aureus* bacteremia was unidentified. Empirical treatment with cefazoline was replaced to vancomycin within 72 h, and the patient was cured.

Both MRSA isolates belonged to ST72 and *agr*-type I. They were negative for both Panton-Valentine leukocidin and toxic shock syndrome toxin (Table 1). The *spa*-types of E16SA093 and F17SA003 were t324 and t2460, respectively. Among the 10 antimicrobials tested, the E16SA093 isolate was resistant only to cefoxitin, while F17SA003 was resistant not only to cefoxitin but also to erythromycin and clindamycin.

**Table 1:** ST72 MRSA isolates harboring the ΨSCC*mec*_E16SA093_ element.

Isolate	Year of isolation	Infection type	Clinical details/sex	Antimicrobial resistance pattern^a^	Antimicrobial resistance gene^b^	Virulence-associated gene^c^	*spa*^d^	*pvl*^e^	TSST-1^e^	*agr*
E16SA093	2016	CA	Bloodstream infection originated from bone infection/female	FOX	*mecA, aadD, blaZ*	*seo, sem, sei, seu, sen, seg, lukE, lukD, aur, splA, splB, hlgB, hlgC, hlgA, hlb, sak, scn*	t324	ND	ND	I
F17SA003	2017	HA	Bloodstream infection of unspecified origin of infection/male	FOX, EM, CLN	*mecA, aadD* *blaZ*^f^	*seo, sem, sei, seu, sen, seg, lukE, lukD, aur, splA, splB, hlgB, hlgC, hlgA, hlb, sak, scn*	t2460	ND	ND	I

CA, community-associated; HA, healthcare-associated; *spa*, staphylococcal protein A; *pvl*, Panton-Valentine leukocidin; TSST-1, toxic shock syndrome toxin; *agr*, accessory gene regulator.

a The antimicrobial resistance was determined against a panel of 10 antistaphylococcal agents, including cefoxitin (FOX), erythromycin (EM), clindamycin (CLN), quinupristin/dalfopristin, cotrimoxazole, mupirocin, vancomycin, teicoplanin, linezolid, and tigecycline.

b The acquired antimicrobial resistance gene was searched for against the database of ResFinder.

c The virulence-associated gene was searched for against the database of VirulenceFinder.

d The *spa* type t324 was 07-23-12-12-17-20-17-12-12-17, and the *spa* type t2460 was 26-17-34-34-17-20-17-17-17-16.

e ND, Not detected.

f Two copies of the *blaZ* gene were identified in the F17SA003 chromosome.

### Genome Sequencing and Identification of the Novel ΨSCC*mec*_E16SA093_

The *de novo* assembly of the genome resulted in a 2,767,631,390-bp circularized chromosome, including 2,564 assigned CDSs, 60 tRNAs, and 19 rRNAs for E16SA093, and a 2,849,947,596-bp circularized chromosome, including 2,546 CDSs, 60 tRNAs, and 19 rRNAs for F17SA003. The overall GC contents were 32.9% for both. No plasmid was identified. Acquired genetic elements by both chromosomes were alike, including two intact *Staphylococcus* prophages (44.1-kb ϕNM3 and 41.2-kb Sap26), 17 virulence factors, and three antimicrobial resistance genes, with an extra copy of *blaZ* for F17SA003. No known heavy metal resistance genes were identified for either.

**Figure 1:**
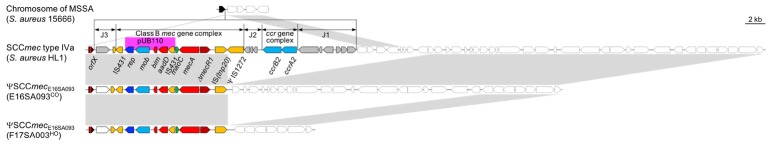
Genomic structures and environments of the ΨSCC*mec*_E16SA093_ element in comparison with the SCC*mec* type IVa in MRSA HL1 (Chen et al., 2013) (GenBank accession, CP003979) and *orfX* in MSSA 15666 (GenBank accession, EU272085). The conserved region in the gray box showed a resemblance of >99% nt identity. Filled arrows represented the components consisting of the SCC*mec* element. The open arrows indicated the presence of chromosomal genes in *S. aureus*. Arrows in black, *orfX*; red, resistance genes; yellow, transposase; blue, replication origin; sky blue, recombinase; gray, hypothetical coding sequence.

For the SCC*mec* element, a class B *mec* gene complex lacking the ΨIS*1272* upstream from the *mecA* gene was identified, and neither the *ccr* gene nor any putative site-specific serine recombinase gene was identified elsewhere in the chromosome (Figure 1). The ΨSCC*mec*, designated as ΨSCC*mec*_E16SA093_, resembled a SCC*mec* type IV, which is common in MRSA ST72. When compared with the genome of HL1 that is a CA ST72-MRSA-IV recovered in South Korea before 2010 (Chen et al., 2013), a 12.6-kb region was deleted from the SCC*mec* type IV element, and 7.0- and 31.9-kb chromosomal DNA region was deleted in the E16SA093 and F17SA003, respectively. The Ccr recombinase involves the site-specific integration/excision of SCC*mec* elements (International Working Group on the Classification of Staphylococcal Cassette Chromosome, 2009), and the CcrA2/CcrB2 in the SCC*mec* IV is targeting the *attB* site at the *orfX* gene (Wang and Archer, 2010). The ΨSCC*mec*_E16SA093_ was indeed integrated exactly at *attB*, suggesting the subsequent elimination of the *ccrA2*/*ccrB2* genes from the SCC*mec* IV element after the integration event. As the ΨIS*1272* was absent, IS-associated recombination was suspected. However, no palindromic sequences were observed in either end of the deleted 19.7- and 44.5-kb DNA fragments targetable by IS*1272*, with an insertion sequence involved in the structure-dependent transposition or stem-loop replacement (Wan et al., 2017).

### Epidemiology of ΨSCC*mec*

The fitness benefit of the *ccr-*gene-loss from SCC*mec*, resulting in an inherent *mecA* in the chromosome, has never been assessed, while spontaneous *mecA*-gene-loss in the absence of selective pressure, driven by the huge biological cost of gene expression, has been demonstrated (Noto et al., 2008). The furnished *mecA* gene could provide advantages to MRSA in the beta-lactam-abundant habitat, such as the clinical settings, suggesting a course of adaptive evolution for MRSA. While ΨSCC*mec* is occasionally identified in MRSA (Chen et al., 2010), methicillin-resistant coagulase-negative staphylococci (MRCNS) carrying the element is much more common (Perreten et al., 2013; Shore and Coleman, 2013). The speculation of MRCNS to be a reservoir of SCC*mec* (Archer et al., 1994), in the MRSA is inspiring.

In this study, we evaluated MRSA ST72 isolates carrying ΨSCC*mec*_E16SA093_, which was likely being fabricated from the SCC*mec* type IV. Excised portions of the chromosomes differed in size, and the event likely occurred independently, indicating that the clonal dissemination of ST72-MRSA-ΨSCC*mec*_E16SA093_ has not yet been occurred. The immobile *mecA* gene could make the MRSA fit the antimicrobial-abundant habitat, even though the *mecA* gene expression is known to be costly. Further study of the molecular mechanisms driving *ccr* gene loss is needed, and dissemination of the clone should be surveilled.

## Ethics Statement

The research, which has no involvement of human subject but the clinical isolates, does meet the exempt category without approval from Ethics Committee on Human Research of the Health Ministry in South Korea and the study design has not been reviewed by the committee.

## Author Contributions

HL and SJ conceived the study. E-JY, HL, and SJ analyzed the data. E-JY and SJ wrote the manuscript. DK, JoS, and JeS collected the strains.

### Conflict of Interest Statement

The authors declare that the research was conducted in the absence of any commercial or financial relationships that could be construed as a potential conflict of interest.

## Footnotes

^1^http://pubmlst.org/saureus

^2^http://www.spaserver.ridom.de

^3^http://www.ncbi.nlm.nih.gov/books/NBK174280

^4^http://blast.ncbi.nlm.nih.gov

^5^https://cge.cbs.dtu.dk/services/ResFinder

^6^https://cge.cbs.dtu.dk/services/VirulenceFinder

^7^http://phaster.ca
